# SR-BI in Bone Marrow Derived Cells Protects Mice from Diet Induced Coronary Artery Atherosclerosis and Myocardial Infarction

**DOI:** 10.1371/journal.pone.0072492

**Published:** 2013-08-13

**Authors:** Ying Pei, Xing Chen, Dina Aboutouk, Mark T. Fuller, Omid Dadoo, Pei Yu, Elizabeth J. White, Suleiman A. Igdoura, Bernardo L. Trigatti

**Affiliations:** 1 Department of Biochemistry and Biomedical Sciences and Thrombosis and Atherosclerosis Research Institute, McMaster University, Hamilton, Ontario, Canada; 2 Department of Biology and Pathology and Molecular Medicine, McMaster University, Hamilton, Ontario, Canada; King’s College London School of Medicine, United Kingdom

## Abstract

SR-BI deficient mice that are also hypomorphic for apolipoprotein E expression develop diet induced occlusive coronary artery atherosclerosis, myocardial infarction and early death. To test the role of SR-BI in bone marrow derived cells, we used bone marrow transplantation to generate SR-BI-null; apoE-hypomorphic mice in which SR-BI expression was restored solely in bone marrow derived cells. SR-BI-null; apoE-hypomorphic mice were transplanted with SR-BI^+/+^apoE-hypomorphic, or control, autologous SR-BI-null; apoE-hypomorphic bone marrow. Four weeks later, mice were fed a high-fat, high-cholesterol, cholate-containing diet to induce coronary artery atherosclerosis. Mice transplanted with autologous bone marrow developed extensive aortic atherosclerosis and severe occlusive coronary artery atherosclerosis after 4 weeks of feeding. This was accompanied by myocardial fibrosis and increased heart weights. In contrast, restoration of SR-BI expression in bone marrow derived-cells reduced diet induced aortic and coronary artery atherosclerosis, myocardial fibrosis and the increase in heart weights in SR-BI-null; apoE-hypomorphic mice. Restoration of SR-BI in bone marrow derived cells did not, however, affect steady state lipoprotein cholesterol levels, but did reduce plasma levels of IL-6. Monocytes from SR-BI-null mice exhibited a greater capacity to bind to VCAM-1 and ICAM-1 than those from SR-BI^+/+^ mice. Furthermore, restoration of SR-BI expression in bone marrow derived cells attenuated monocyte recruitment into atherosclerotic plaques in mice fed high fat, high cholesterol cholate containing diet. These data demonstrate directly that SR-BI in bone marrow-derived cells protects against both aortic and CA atherosclerosis.

## Introduction

The scavenger receptor class B type I (SR-BI) is a multiligand receptor that binds to high density lipoprotein (HDL) with high affinity and mediates the selective uptake of HDL lipids [1], cellular cholesterol efflux from cells [2] and HDL dependent signaling [3]. SR-BI knockout (KO) mice exhibit defective HDL-dependent reverse cholesterol transport from macrophages, resulting in increased plasma HDL-associated cholesterol and reduced cholesterol in bile [4]–[9]. SR-BI deficiency results in increased atherosclerosis in apoE or LDL receptor KO or diet induced models of atherosclerosis [8], [10]–[14]. Overexpression of SR-BI in livers of mice has the opposite effects, reducing both HDL cholesterol levels and atherosclerosis [15]–[20].

SR-BI/apoE double KO mice rapidly (within 6 weeks of age) develop occlusive coronary artery (CA) atherosclerosis and exhibit extensive myocardial fibrosis, ECG abnormalities, cardiac enlargement, reduced heart function (ejection fraction, contractility and relaxation) and early death [10], [21]–[23]. The SR-BI^−/−^apoE-R61^h/h^ (hypomorphic) mouse is a related model [14] that lacks SR-BI and has a targeted mutation in the apoE gene that encodes a Thr61Arg point mutant form of apoE that exhibits reduced clearance [24], [25]. Furthermore, the apoE expression level is only 2–5% of normal level due to the insertion of a neomycin resistance gene cassette in the third intron of the apoE gene [25]. When fed a normal low fat, low cholesterol diet, SR-BI^−/−^apoE-hypomorphic mice develop little aortic or CA atherosclerosis. However, when fed a high fat, high cholesterol diet containing cholate (HFCC diet), these mice rapidly develop aortic and CA atherosclerosis and cardiac morphological, conductance and functional abnormalities that are strikingly similar to those seen in SR-BI/apoE double KO mice [14]. These mice do not survive beyond 3.5–5 weeks of the feeding period [14]. These SR-BI/apoE dKO mice and HFCC diet fed SR-BI^−/−^apoE-hypomorphic mice exhibit many features of human coronary heart disease (CHD), stemming from occlusive CA disease.

SR-BI is expressed in several cell types relevant to atherosclerosis development, including hepatocytes, bone marrow derived cells (monocytes, macrophages, dendritic cells and platelets) and vascular wall cells (endothelial and smooth muscle cells) [26]–[32]. SR-BI in hepatocytes mediates selective HDL lipid uptake and clearance from plasma, driving RCT [4]–[7], [33]. SR-BI also plays an important role in the vascular wall. Transplantation of bone marrow (BM) stem cells from donor mice lacking SR-BI into recipient LDL receptor KO, apoE KO or wild type mice increases their susceptibility to atherosclerosis [11], [34], [35]. Conversely, transplantation of BM from wild type mice into lethally irradiated SR-BI/apoE dKO mice rescues their survival and reduces aortic and CA atherosclerosis [36]. Expression of apoE in BM-derived cells has previously been shown to protect against aortic atherosclerosis in apoE KO mice [37], [38]. However, the effect of specifically restoring normal SR-BI expression in BM derived cells on atherosclerosis in SR-BI-deficient mice is not known.

In this study, we tested the effects of restoring SR-BI alone in BM-derived cells on the development of diet induced aortic and CA atherosclerosis, heart size and cardiac fibrosis in SR-BI^−/−^apoE-hypomorphic mice. We demonstrate that restoration of normal SR-BI expression in BM-derived cells protected against diet induced aortic and CA atherosclerosis, myocardial infarction and increases in heart weights in SR-BI^−/−^apoE-hypomorphic mice, without substantially affecting plasma or lipoprotein cholesterol levels. Instead our data point to a role for SR-BI in bone marrow derived cells in attenuating the recruitment of monocytes into atherosclerotic plaques.

## Results

### Restoration of SR-BI in BM derived cells protects SR-BI^−/−^apoE-hypomorphic mice against diet induced atherosclerosis in aortas and coronary arteries

It has previously been shown that transplantation with bone marrow from wild type donors protects SR-BI/apoE double KO mice from spontaneous occlusive coronary artery atherosclerosis and accelerated aortic sinus atherosclerosis [36]. The spontaneous CHD that develops in SR-BI/apoE double KO mice mirrors the diet induced CHD in SR-BI^−/−^apoE-hypomorphic mice [10], [14]. To determine the contribution of SR-BI in BM derived cells to the protection against aortic sinus and coronary artery atherosclerosis we tested if restoration of normal SR-BI expression in BM-derived cells by transplantation of SR-BI^−/−^apoE-hypomorphic mice with BM from SR-BI^+/+^apoE-hypomorphic donors (SR-BI^+/+^apoE-hypomorphic→SR-BI^−/−^apoE-hypomorphic) might affect the development of atherosclerosis in aortic sinus and coronary arteries. SR-BI^−/−^apoE-hypomorphic→ SR-BI^−/−^apoE-hypomorphic mice were used as controls. All mice used in this study were of the apoE-hypomorphic (apoE-R61^h/h^) genotype and were either SR-BI^+/+^ or SR-BI^−/−^. For simplicity, we refer to the mice solely by the SR-BI genotype. Thus, SR-BI^+/+^→SR-BI^−/−^ refers to SR-BI^−/−^apoE-hypomorphic mice that have been transplanted with BM from SR-BI^+/+^apoE-hypomorphic donors. Likewise, SR-BI^−/−^→SR-BI^−/−^ mice refer to control SR-BI^−/−^apoE-hypomorphic mice that have been transplanted with BM from SR-BI^−/−^apoE-hypomorphic donors.

**Figure 1:**
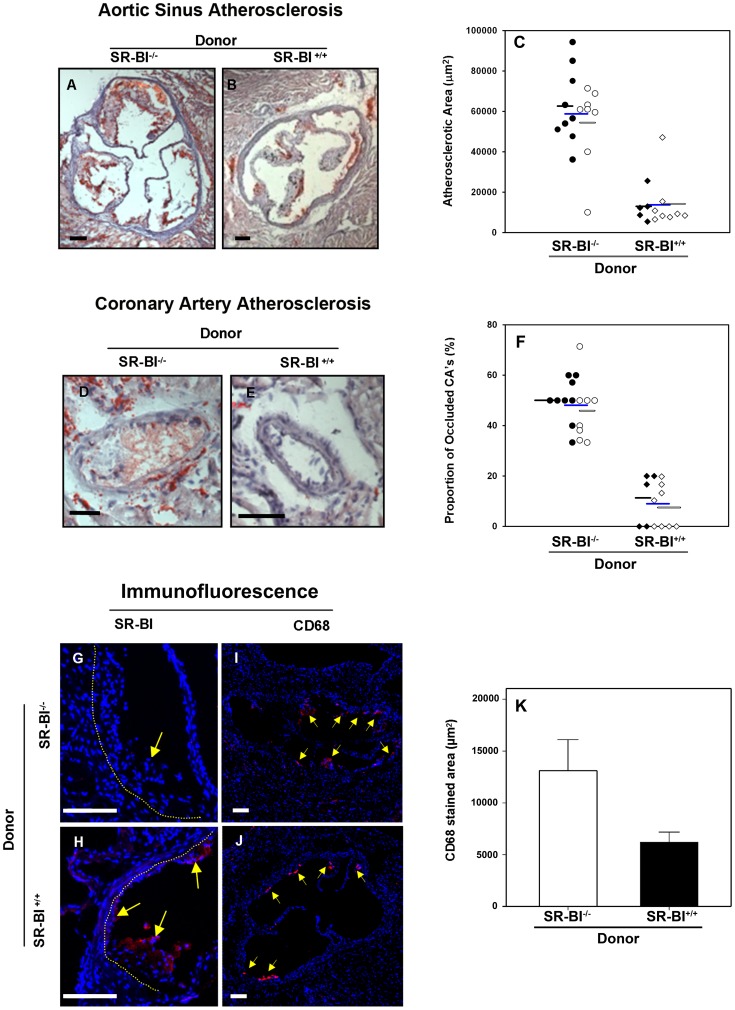
Restoration of SR-BI expression in BM derived cells by BM transplantation reduces diet induced atherosclerosis in SR-BI-null/apoE-hypomorphic mice. SR-BI^−/−^apoE-hypomorphic mice, at 10 weeks of age, were transplanted with BM from either control SR-BI^−/−^ or SR-BI^+/+^ mice to generate SR-BI^−/−^ → SR-BI^−/−^ (circles) and SR-BI^+/+^→ SR-BI^−/−^ mice (diamonds). BM transplanted mice were fed a high fat, high cholesterol, cholate containing (HFCC) diet for four weeks. **A–C:** Aortic sinus atherosclerosis. Representative oil red O and hematoxylin stained sections of the aortic sinus are shown in panels **A** and **B** for mice transplanted with BM from SR-BI^−/−^ or SR-BI^+/+^ donors. Scale bars = 100 µm. **C.** Atherosclerotic plaque sizes were quantified for n = 17 SR-BI^−/−^ → SR-BI^−/−^ and n = 13 SR-BI^+/+^→ SR-BI^−/−^ mice. Donor BM is indicated. P<0.001 by the Mann Witney rank sum test. **D–F:** Coronary artery atherosclerosis. Representative images of oil red O and hematoxylin stained coronary artery sections are shown in panels **D** and **E.** Scale bars = 50 µm. **F.** Coronary arteries in heart sections were scored as “occluded” if they contained raised atherosclerotic plaques and the proportions of occluded coronary arteries are plotted. Group sizes are as for Panel C. P = 3×10^−9^ by Student’s T-test and P<0.001 by the Mann Witney rank sum test. For panels C and F, male and female mice are indicated by closed and open symbols respectively. Averages are indicated by bars: black bars  =  males, open bars  =  females, blue bars  =  pooled males and females. Data for males vs females were not statistically significantly different. Immunostaining (red) for SR-BI (G, H) or CD68 (**I, J**) in atherosclerotic plaques from a separate group of female SR-BI^−/−^ → SR-BI^−/−^ mice (**G, I**) and SR-BI^+/+^→ SR-BI^−/−^ mice (**H, J**) fed the HFCC diet for 18 days. Representative images are shown. Yellow arrow in G points to SR-BI negative cells; yellow arrows in H-J indicate cells positive for SR-BI or CD68. Nuclear DNA was stained with DAPI. Scale bars = 100 µm. **K.** Quantification of CD68 staining area in n = 6 mice per group (all females). Data are means ± standard errors. P = 0.039 by Student’s T-test.

Four weeks after BM transplantation, donor stem cell engraftment was confirmed by PCR genotyping of blood cell DNA (not shown). Transplanted mice were fed a diet containing 15% fat, 1.25% cholesterol and 0.5% cholate (HFCC diet). Krieger and co-workers previously reported that SR-BI^−/−^apoE-hypomorphic mice did not survive beyond 4 weeks of feeding on this diet [14]. Therefore, the mice were euthanized at 4 weeks of feeding and atherosclerosis in the aortic sinus and coronary arteries was analyzed. Consistent with findings reported previously for SR-BI^−/−^apoE-hypomorphic mice [14], the HFCC diet-fed SR-BI^−/−^→ SR-BI^−/−^ mice developed significant atherosclerosis in the aortic sinus and extensive occlusive atherosclerosis in coronary arteries. The mean cross sectional areas of atherosclerotic plaques in the aortic sinus of male and female HFCC diet fed SR-BI^−/−^→SR-BI^−/−^ mice were 63,000±7,000 and 54,000±7,000 µm^2^, and were not statistically significantly different; when males and females were pooled the mean was 59,000±5,000 µm^2^ (Figure 1A, C). Furthermore, male and female mice exhibited means of 50±3 and 45±4% (not significantly different; mean of pooled data, 48±3%) of coronary arteries in heart sections that were at least partially occluded with lipid rich atherosclerotic plaques (Figure 1D, F). In contrast, SR-BI^+/+^ → SR-BI^−/−^ mice, fed the same diet, developed substantially reduced levels of atherosclerosis over the same time period. The mean cross sectional areas of atherosclerotic plaques in the aortic sinus of male and female HFCC diet fed SR-BI^+/+^→SR-BI^−/−^ mice were 14,200±5,000 and 13,000±3,000 µm^2^, and were not statistically significantly different; when males and females were pooled, the mean was 13,800±3,000 µm^2^ (Figure 1 B, C). Furthermore, male and female SR-BI^+/+^→SR-BI^−/−^ mice exhibited only approximately 11±5 and 7.5±3% (not significantly different; mean of pooled data was 9±3%) of coronary arteries in heart sections that were at least partially occluded with lipid rich atherosclerotic plaques (Figure 1 E, F). Therefore, transplantation of bone marrow from SR-BI^+/+^ donors resulted in reductions in diet induced aortic sinus and coronary artery atherosclerosis of 75 and 80%, respectively. Indirect immunofluorescence staining for SR-BI and for the macrophage marker, CD68 was carried out in a separate set of transplanted female mice fed the HFCC diet for 18 days. This verified that SR-BI was indeed expressed in cells in atherosclerotic plaques in the SR-BI^+/+^→SR-BI^−/−^ mice but not in control SR-BI^−/−^→SR-BI^−/−^ mice (Figure 1 G, H). Staining for CD68 is shown in representative sections in Figure 1I and J, and quantified in sections from 6 mice per group in panel K. This revealed significantly reduced macrophage content in plaques in the aortic sinus from SR-BI^+/+^→SR-BI^−/−^ compared to control SR-BI^−/−^→SR-BI^−/−^ mice.

**Figure 2:**
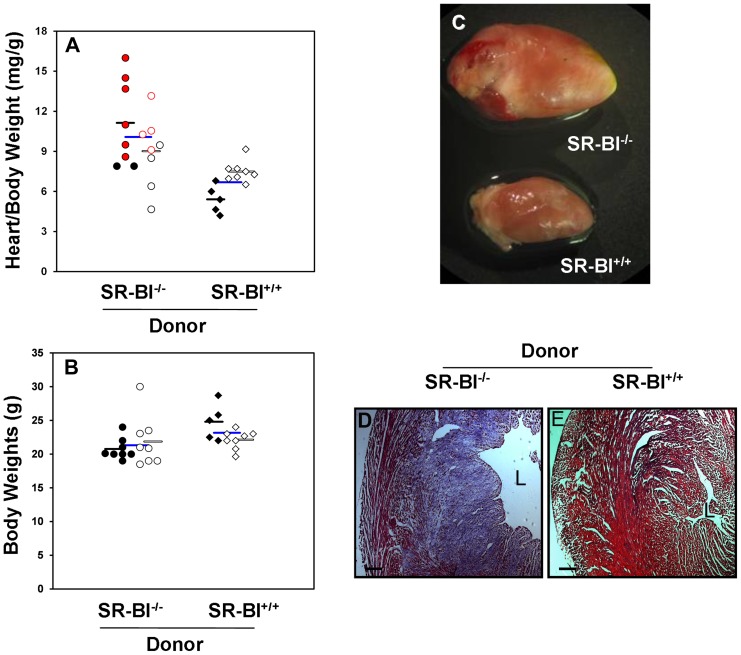
Restoration of SR-BI expression in BM derived cells attenuates diet induced increases in heart size and cardiac fibrosis in SR-BI-null/apoE-hypomorphic mice. **A.** Ratios of heart weight to body weight (HW:BW) are plotted for SR-BI^−/−^ → SR-BI^−/−^ mice (circles) and SR-BI^+/+^ → SR-BI^−/−^ mice (diamonds) fed the HFCC diet for 4 weeks. Red symbols identify those SR-BI^−/−^ → SR-BI^−/−^ mice which appeared to be in poor health or were moribund at collection. Closed symbols denote males and open symbols denote females. Horizontal bars indicate the mean heart/body weight ratios (black bars  =  males, open bars  =  females, blue bars  =  pooled males and females). Data passed the Shapiro-Wilk test for normality and was analyzed by the Student’s T test; P<0.0006 for SR-BI^−/−^ → SR-BI^−/−^ vs SR-BI^+/+^ → SR-BI^−/−^ mice. **B.** Body weights from the same mice as in A are plotted for male (closed symbols) and female (open symbols) SR-BI^−/−^ → SR-BI^−/−^ (circles) and SR-BI^+/+^ → SR-BI^−/−^ (diamonds). Bars denote average body weights: black bars  =  males, open bars  =  females; blue bars  =  pooled male + female mice. Differences in body weights between males (P = 0.006) and when males and females were pooled (P = 0.03) were significant, but females were not, by Student’s T test. **C.** Appearance of hearts from SR-BI^−/−^ → SR-BI^−/−^ and SR-BI^+/+^ → SR-BI^−/−^ mice after 4 weeks of HFCC diet feeding. **D–E.** Trichrome staining of cross sections of hearts from SR-BI^−/−^ → SR-BI^−/−^ (D) and SR-BI^+/+^ → SR-BI^−/−^ mice (E) after 4 weeks of HFCC diet feeding. Healthy myocardium appears red while collagen-rich fibrotic areas appear blue. L indicates the lumen of the left ventricle. Representative images are shown in C–E. Scale bars  =  300 µm.

Male SR-BI^−/−^→SR-BI^−/−^ mice fed the HFCC diet for 4 weeks exhibited relatively high heart/body weight (HW/BW) ratios (11±1 mg/g). In contrast, HW/BW ratios from male SR-BI^+/+^→SR-BI^−/−^ mice fed the HFCC diet for 4 weeks (Fig 2 A) were substantially, and statistically significantly lower (5.4±0.5 mg/g). HW/BW ratios from females tended to be lower for SR-BI^+/+^→SR-BI^−/−^ (7.5±0.3 mg/g) than SR-BI^−/−^→SR-BI^−/−^ (9.0±0.9 mg/g) but this did not reach statistical significance (Fig 2 A). In general, the highest HW/BW ratios were observed in mice that either exhibited outward signs of illness, including ruffled fur, a hunched posture, lack of mobility, and panting prior to euthanasia, or mice which were found moribund and immediately euthanized (Figure 2A, red symbols). On the other hand, mice which appeared to be outwardly healthy (Figure 2A, black symbols) exhibited lower HW/BW ratios. These differences in HW/BW ratios (50% reduction for males; 37% reduction when males and females were pooled) were not driven by the more modest differences in body weights (Figure 2B; 20.8± 1.6 g versus 24.8±2.7 g, for male; 21.9±3.8 g versus 22.1±1.4 g for female SR-BI^−/−^→SR-BI^−/−^ versus SR-BI^+/+^→SR-BI^−/−^ mice, respectively). Furthermore, many of the hearts from SR-BI^−/−^→SR-BI^−/−^ mice appeared large and had pale discolorations suggestive of scarring (Figure 2C). Their appearance was consistent with cardiac enlargement previously described for un-transplanted HFCC diet fed SR-BI^−/−^apoE-hypomorphic mice as well as for normal diet fed SR-BI^−/−^apoE^−/−^ mice [10], [14]. In contrast, hearts from SR-BI^+/+^→SR-BI^−/−^ mice were visibly smaller and did not exhibit evidence of surface scarring (Figure 2C). Transverse sections of hearts from control SR-BI^−/−^→SR-BI^−/−^, stained with Mason’s Trichrome revealed extensive regions which were rich in collagen (blue staining) with very little intact myocardium (red staining) (Figure 2D), consistent with the extensive cardiac fibrosis which has previously been described for HFCC diet fed SR-BI^−/−^apoE-hypomorphic mice [14]. In contrast, hearts from SR-BI^+/+^→SR-BI^−/−^ mice fed the HFCC diet for the same period of time did not exhibit any evidence of collagen-rich fibrotic areas (Figure 2E). Together, these data demonstrate that the restoration of SR-BI in BM derived cells protects SR-BI^−/−^apoE-hypomorphic mice from diet induced aortic sinus and occlusive coronary artery atherosclerosis, cardiac enlargement and cardiac fibrosis.

### Effects of SR-BI in BM derived cells on plasma lipids and markers of inflammation

**Figure 3:**
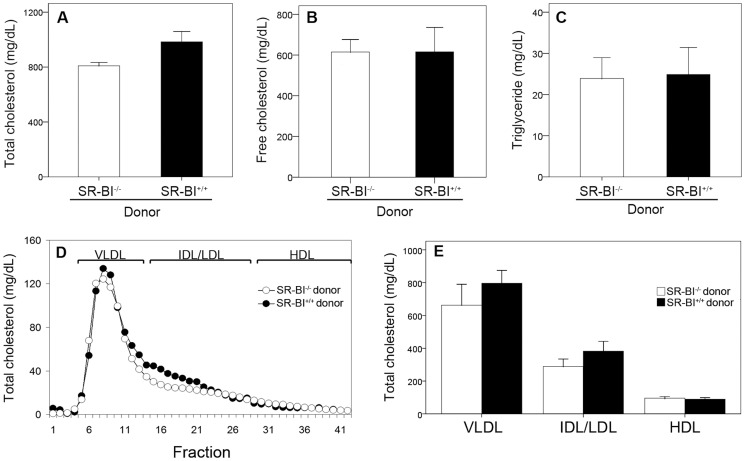
Restoration of SR-BI expression in BM derived cells did not alter lipids in HFCC diet fed SR-BI-null/apoE-hypomorphic mice. **A.** Total cholesterol, **B.** unesterified (free) cholesterol and **C.** triglycerides were measured in plasma collected from fasted SR-BI^−/−^ → SR-BI^−/−^ (white bars) and SR-BI^+/+^ → SR-BI^−/−^ mice (black bars) fed the HFCC diet for 3 weeks (n = 6 mice/group). **D.** Averaged lipoprotein total cholesterol profiles from n = 10 SR-BI^−/−^ → SR-BI^−/−^ (white symbols) and SR-BI^+/+^ → SR-BI^−/−^ mice (black symbols) fed the HFCC diet for 3 weeks. Fractions corresponding to the elution of purified human VLDL, IDL/LDL and HDL are shown at the top. **E.** Total cholesterol fractions containing VLDL-sized (1–14), IDL/LDL-sized (15–28) and HDL-sized lipoproteins (29–42) from the lipoprotein total cholesterol profiles of individual mice were determined. Averages ± standard errors are shown (n = 10 mice/group). No statistically significant differences in values from SR-BI^−/−^ → SR-BI^−/−^ (white bars) and SR-BI^+/+^ → SR-BI^−/−^ mice (black bars) were detected.

Analysis of plasma lipids revealed that there were no significant differences in plasma total cholesterol, free cholesterol or triglyceride levels between SR-BI^−/−^→SR-BI^−/−^ and SR-BI^+/+^→SR-BI^−/−^ mice fed the HFCC diet for 3 weeks (Figure 3A–C). As previously reported for un-transplanted SR-BI^−/−^apoE-hypomorphic mice fed a similar diet for a similar period of time, the majority of plasma cholesterol was unesterified, or free cholesterol (Figure 3B), and plasma triglyceride levels were very low (Figure 3C) compared to plasma total cholesterol (Figure 3A) [14]. The distribution of cholesterol across different lipoproteins was analyzed after size separation of plasma lipoproteins by FPLC and analysis of the cholesterol content of each fraction. Averaged lipoprotein cholesterol profiles are shown in Figure 3D and the amounts of cholesterol in fractions corresponding to very low density (VLDL)-, intermediate and low density (IDL/LDL)- and high density (HDL)-sized lipoproteins are shown in Figure 3E. These analyses revealed that there were no statistically significant differences in cholesterol associated with VLDL, IDL/LDL or HDL sized lipoproteins between the two groups of mice. Thus restoration of SR-BI expression in BM derived cells did not affect steady state lipoprotein cholesterol levels in HFCC diet fed SR-BI^−/−^apoE-R61^h/h^ mice.

**Figure 4:**
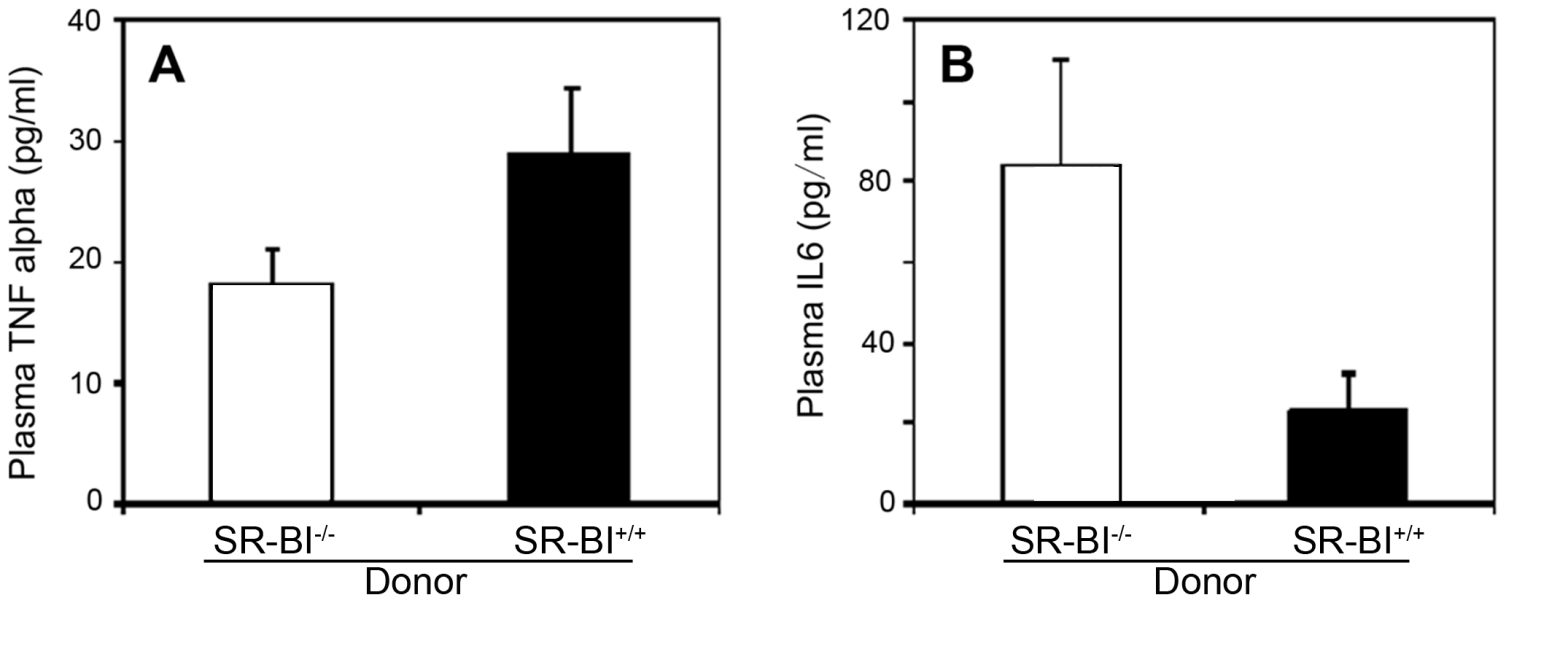
Effects of restoring SR-BI expression in BM derived cells on plasma levels of TNFα and IL-6 in HFCC diet fed SR-BI-null/apoE-hypomorphic mice. **A.** TNFα and **B.** IL-6 levels in plasma from control SR-BI^−/−^ → SR-BI^−/−^ (white bars) and SR-BI^+/+^ → SR-BI^−/−^ mice (black bars) after 3 weeks of HFCC diet feeding. Shown are mean levels for n = 8 mice per group. Error bars correspond to standard errors. **A.** TNFα levels were not statistically significantly different. **B.** IL-6 levels were statistically significantly different. P = 0.028. Statistical analysis was by the Mann-Whitney rank sum test.

Levels of TNFα and IL-6 in plasma were also analyzed (Figure 4A, B). TNFα levels appeared to be slightly higher in HFCC diet fed SR-BI^+/+^→SR-BI^−/−^ mice than in SR-BI^−/−^→SR-BI^−/−^ mice, although the increase did not reach statistical significance (Figure 4A). In contrast, plasma IL-6 levels were substantially and significantly lower in SR-BI^+/+^→SR-BI^−/−^ mice than in SR-BI^−/−^→SR-BI^−/−^ mice fed the HFCC diet for 3 weeks (Figure 4B; 23±9 versus 84±26 pg/ml, respectively). This >70% reduction in circulating IL-6 levels suggests that restoration of SR-BI expression in BM derived cells may attenuate at least some pro-atherogenic inflammatory pathways.

**Figure 5:**
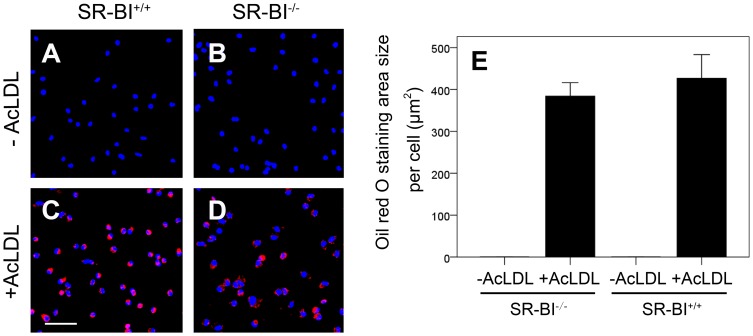
Knockout of SR-BI in macrophages does not affect AcLDL driven foam cell formation. Resident peritoneal macrophages collected from SR-BI^+/+^apoE-hypomorphic or SR-BI^−/−^apoE-hypomorphic mice were cultured in the absence or presence of 100 µg/ml AcLDL for 24 hrs, fixed and stained with oil red O (red) and DAPI (blue). **A**–**D**: Representative fluorescence images. Scale bar  =  100 µm. **E**. Quantification of lipid accumulation as the oil red O staining intensity normalized to the number of DAPI stained nuclei per field of view. Five fields of view were analyzed per sample. Data are averages ± standard errors of triplicate samples. The experiment was performed three times and a representative result is shown. Oil red O staining intensities for AcLDL treated versus untreated cells were statistically significantly different but staining intensities for AcLDL treated SR-BI^+/+^ and SR-BI^−/−^ cells were not (determined by Student’s T test).

Macrophage foam cells constitute the major cellular component of atherosclerotic plaques. We therefore tested whether SR-BI expression affected macrophage foam cell formation. Resident peritoneal macrophages prepared from SR-BI^+/+^apoE-hypomorphic and SR-BI^−/−^ apoE-hypomorphic mice were cultured in the absence or presence of AcLDL and stained with oil red O (Figure 4). Macrophages from neither the SR-BI^+/+^ nor the SR-BI^−/−^ mice showed evidence of oil red O stained lipid droplets when they were cultured in the absence of AcLDL (Figure 5A, B). Both accumulated oil red O stained lipid droplets to a similar extent when cultured in the presence of AcLDL (Figure 5C, D, quantified in E). Similar results were obtained when macrophages were prepared from mice that had been fed the HFCC diet for 2 weeks (not shown). Therefore, SR-BI in macrophages does not appear to directly affect the degree of foam cell formation, at least under the *in vitro* assay conditions we employed.

### Monocytes from SR-BI KO mice exhibit increased capacity to bind ICAM-1 and VCAM-1 and restoration of SR-BI in BM derived cells reduced monocyte recruitment into atherosclerotic plaques

**Figure 6:**
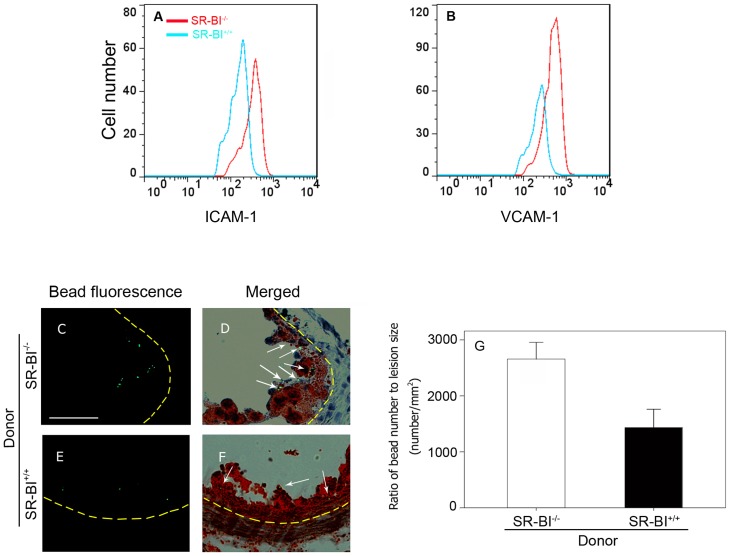
SR-BI attenuates monocyte recruitment. **A.** ICAM-1 binding, **B.** VCAM-1 binding, by monocytes from SR-BI^+/+^apoE-hypomorphic (blue) or SR-BI^−/−^apoE-hypomorphic mice (red) as determined by flow cytometry. Representative histograms of 4 replicates are shown. **C**–**G**: *In vivo* recruitment of latex bead marked monocytes into atherosclerotic plaques. Fluorescence images in dark-field (C and E) or overlayed onto brightfield images (D and F) of oil red O stained aortic sinus atherosclerotic plaques from SR-BI^−/−^ → SR-BI^−/−^ (C, D) and SR-BI^+/+^ → SR-BI^−/−^ mice (E, F). Arrows in D and F indicate the positions of green fluorescent latex bead marked cells. Scale bar  =  50 µm. G. Quantification of fluorescent bead marked cells in atherosclerotic plaques (normalized to plaque sizes). Data are averages ± standard errors. N = 4 mice per group. P = 0.039 by Student’s T test.

ICAM-1 and VCAM-1, expressed on endothelial cells play critical roles in the capture of circulating monocytes and their recruitment into growing atherosclerotic plaques [39]. We therefore tested if monocytes from SR-BI^−/−^apoE-hypomorphic and SR-BI^+/+^apoE-hypomorphic mice differed in their abilities to bind to ICAM-1 or VCAM-1. To do this we used soluble recombinant ICAM-1 or VCAM-1 fused to human IgG Fc, and monitored binding using a flow cytometry assay in conjunction with an anti-human IgG antibody. Monocytes from SR-BI^−/−^ mice showed increased binding to both ICAM-1 (Figure 6A) and VCAM-1 (Figure 6B) compared to monocytes from SR-BI^+/+^ mice. We obtained equivalent results when we repeated the experiment using monocytes from mice fed the HFCC diet for 2 weeks (not shown). These findings suggested that monocytes from SR-BI^−/−^ mice have increased capacity for recruitment into atherosclerotic plaques and that the reduced atherosclerosis in SR-BI^+/+^→SR-BI^−/−^ mice compared to the SR-BI^−/−^→SR-BI^−/−^ mice may be because restoration of SR-BI expression in BM derived cells reduces monocyte recruitment into developing atherosclerotic plaques. To test this, we utilized a well characterized *in vivo* assay for monocyte recruitment into atherosclerotic plaques [40] in which fluorescent latex beads were injected intravenously to mark circulating monocytes which phagocytose the latex beads. For this study, SR-BI^+/+^→SR-BI^−/−^ and control SR-BI^−/−^→SR-BI^−/−^ mice were fed the HFCC diet for 17 days prior to i.v. injection with fluorescent latex beads. Twenty four hours later, the mice were euthanized and atherosclerotic plaques in the aortic sinus were stained with oil red O and visualized by light and fluorescence microscopy (Figure 6C-F). We found that SR-BI^+/+^→SR-BI^−/−^ mice accumulated approximately 40% fewer latex bead marked cells in atherosclerotic plaques than did control SR-BI^−/−^→SR-BI^−/−^ mice (Figure 6G), even though the lesion sizes at this stage (18 d of HFCC diet feeding) were similar between the two groups (data not shown). Therefore, restoration of SR-BI expression in BM derived cells in SR-BI-deficient mice attenuated monocyte recruitment.

## Discussion

SR-BI^−/−^apoE^−/−^ mice develop spontaneous and SR-BI^−/−^apoE-hypomorphic mice develop high fat, high cholesterol diet-induced accelerated aortic sinus and occlusive coronary artery atherosclerosis that is accompanied by cardiac fibrosis and enlargement, cardiac conductance and function abnormalities and early death within weeks [10], [14], [21]–[23]. Transplantation of SR-BI^−/−^apoE^−/−^ mice with BM from wild type donors was previously shown by others to reduce the development of aortic atherosclerosis and rescue them from early death, presumably due to a reduction in occlusive CA atherosclerosis [36]. However the effect of restoring only SR-BI expression in BM derived cells has not been tested. Using a BM transplantation approach, we now demonstrate that selective restoration of SR-BI expression in BM derived cells is able to protect otherwise SR-BI-deficient and apoE-hypomorphic mice from diet induced atherosclerosis in the aortic sinus and coronary arteries, and is able to reduce the extent of cardiac fibrosis and cardiac enlargement (as qualitatively assessed by the appearance of the hearts and quantified by the heart/body weight ratios).

This demonstrates that restoring the expression of SR-BI alone in BM-derived cells results in reduced atherosclerosis in otherwise SR-BI-deficient mice. It is consistent with reports from our lab and others that eliminating SR-BI in BM-derived cells in mice that otherwise express SR-BI accelerates atherosclerosis in the aortae [11], [34], [35]. The protective effect of SR-BI in BM-derived cells does not appear to be the consequence of altered overall lipoprotein metabolism since SR-BI^+/+^→SR-BI^−/−^ and control SR-BI^−/−^→SR-BI^−/−^ mice did not have significantly or substantially different levels of plasma or lipoprotein total cholesterol, or plasma free cholesterol or triglycerides when fed the HFCC diet for 3 weeks.

The HFCC diet that we used has been reported to result in systemic increases in inflammatory cytokine levels [41], [42]. Furthermore, we and others have reported that mice lacking SR-BI exhibited increased levels of inflammatory cytokines in plasma compared to SR-BI^+/+^ controls [43], [44]. We therefore tested if restoration of SR-BI in BM derived cells might result in lower levels of circulating inflammatory cytokines. We saw no statistically significant differences in levels of plasma TNFα but saw significantly and substantially reduced levels of plasma IL-6 in SR-BI^+/+^→SR-BI^−/−^ compared to control SR-BI^−/−^→SR-BI^−/−^ mice. Elevated levels of IL-6 have been shown to induce aortic sinus atherosclerosis in apoE KO mice [45]. Despite the elevated levels of IL-6, we did not find any difference in the tendency of macrophages from SR-BI expressing or deficient mice to form foam cells when challenged *in vitro* with AcLDL, suggesting that reduced foam cell formation most likely does not underlie the reduced atherosclerosis we observed in SR-BI^+/+^→SR-BI^−/−^ compared to control SR-BI^−/−^→SR-BI^−/−^ mice.

Monocytes express low levels of SR-BI and levels increase upon differentiation to macrophages (our unpublished data and[29], [46], [47]). HDL has been reported to modulate the activity or expression levels of the integrin subunits CD11b and CD49d on monocytes, via SR-BI[46], [48]. Upon activation, monocyte integrins containing CD11b or CD49d interact with ICAM-1 or VCAM-1 on endothelial cells, mediating monocyte entry into the vessel wall, a key initiating step in the development of atherosclerotic plaques. We found that monocytes from SR-BI deficient (SR-BI^−/−^ apoE-hypomorphic) mice exhibited substantially increased capacity to bind to both ICAM-1 and VCAM-1 *in vitro* compared to monocytes from SR-BI expressing (SR-BI^+/+^apoE-hypomorphic) mice. To explore the *in vivo* consequences of this increased capacity to bind to ICAM-1 and VCAM-1, we used a well characterized assay for recruitment of monocytes into atherosclerotic plaques [40]. Importantly, we analyzed monocyte recruitment into developing atherosclerotic plaques after only 18 days of HFCC diet feeding when atherosclerotic plaques in the aortic sinus were still relatively small. This “fluorescent bead recruitment assay” revealed that there was an approximate 40% reduction in monocyte recruitment into atherosclerotic plaques in SR-BI^+/+^→SR-BI^−/−^ compared to control SR-BI^−/−^→SR-BI^−/−^ mice. This reduced monocyte recruitment is consistent with the reduced capacity of SR-BI^+/+^ monocytes to bind to ICAM-1 and VCAM-1 and is consistent with the previously hypothesized role for monocyte SR-BI in modulating monocyte recruitment [46], [48]. On the other hand it has been proposed that IL-6 promotes monocyte recruitment during chronic inflammation [49], suggesting the possibility that reduced monocyte recruitment in SR-BI^+/+^→SR-BI^−/−^ mice may also be driven by the reduced levels of circulating IL-6.

In conclusion, our data suggest that restoration of SR-BI in BM-derived cells is sufficient to protect against diet-induced aortic sinus and occlusive CA atherosclerosis, and subsequent myocardial infarction and cardiac enlargement in SR-BI^−/−^apoE-hypomorphic mice. All of the control SR-BI^−/−^→SR-BI^−/−^ mice analyzed exhibited coronary artery atherosclerosis but only a subset exhibited increased heart/body weights and cardiac enlargement. This suggests that coronary artery atherosclerosis precedes the cardiac enlargement and is consistent with the notion that cardiac fibrosis and cardiac enlargement are consequences of the extensive, occlusive coronary artery atherosclerosis in this model. In this light, we think it is most likely that the effect of restoration of SR-BI expression in BM derived cells on cardiac fibrosis and cardiac enlargement is primarily due to the reduction in coronary artery atherosclerosis burden, possibly through attenuated monocyte recruitment.

## Materials and Methods

### Mice

All procedures were approved by the McMaster University Animal Research Ethics Board and were in accordance with Canadian Council on Animal Care guidelines. SR-BI^+/−^apoE-hypomorphic mice, originally obtained from Monty Krieger (MIT, Cambridge MA, USA) were bred to generate littermate SR-BI^−/−^apoE-hypomorphic and SR-BI^+/+^apoE-hypomorphic offspring.

### Bone marrow transplantation

Recipient mice (10 weeks old), fed normal chow diets, were exposed to 7Gy of γ-irradiation from a ^137^Cs source using a Gammacel 3000 small animal irradiator. BM (3×10^6^ cells), prepared from the tibias and femurs of donor mice, was injected i.v. 7 Gy was a lethal dose for untreated SR-BI^−/−^apoE-hypomorphic mice and transplanted mice did not survive higher doses of irradiation (data not shown). In some experiments, SR-BI^−/−^apoE-hypomorphic mice were fed, starting at weaning, normal chow diet supplemented with 0.5% probucol [21] until they were used as recipients at 10 weeks of age. These mice were able to tolerate much higher doses of irradiation (up to 13 Gy) and exhibited increased survival of the BM transplantation procedure. Post transplantation, mice received normal chow, soaked with water and antibiotics as described previously [11]. Four weeks post-transplantation, blood was collected and DNA was prepared from blood cells for detection of donor-derived genes by PCR as described previously [11].

### Diet induction of atherosclerosis

Four weeks after BMT, mice were fed a 7.5% cocoa butter (15% total fat), 1.25% cholesterol, 0.5% sodium cholate-containing diet (high fat, cholesterol, cholate, or HFCC diet; Teklad Research Diets) for 4 weeks [14]. Mice were fasted overnight and then euthanized either by CO_2_ asphyxiation or anesthetic overdose. Blood was collected by cardiac puncture and tissues were perfused and prepared for cryosectioning as described previously [8], [10].

### Atherosclerosis and myocardial fibrosis

Atherosclerosis in oil red O stained/hematoxylin counter-stained frozen sections of the aortic sinus was measured as described previously [8]. Coronary artery atherosclerosis was detected in at least 2 oil red O stained transverse sections from different levels of the heart for each mouse. The proportion of coronary artery cross sections containing atherosclerotic plaques (occluded) was determined [10], [23]. Myocardial fibrosis was detected as blue collagen staining using Masson’s Trichrome staining kit (Sigma) [10], [23].

### Immunofluorescence staining

BM transplanted mice were fed the HFCC diet for 3 weeks and euthanized. Aortic sinus sections were separately stained with a rabbit anti-mouse SR-BI (Novus Biologicals, Oakville, ON Canada) or rat anti-CD68 antibody (AbD Serotec, Raleigh, NC USA), followed by incubation with goat anti-rabbit alexa-594 or goat anti-rat alexa 568 secondary antibodies (Invitrogen-Life Technologies Inc, Burlington ON, Canada) and staining with 4',6-diamidino-2-phenylindole (DAPI). Fluorescent images were observed under a Zeiss Axiovert 200 M inverted fluorescence microscope. 8 sections were analyzed per mouse and 6 mice per group were done. CD68 staining was quantified as the area of the plaque staining positive for CD68.

### Analysis of plasma lipids and cytokines

BM transplanted mice were fed the HFCC diet for 21 days, fasted overnight, anesthetized, exsanguinated and euthanized. Plasma was fractionated by gel filtration fast-protein liquid chromatography using an AKTA system with a Tricorn Superose 6 HR10/300 column (GE Biosciences) as previously described [7], [8], [11]. Coupled enzymatic assay kits were used to analyze plasma and lipoprotein total cholesterol levels (Infinity Cholesterol Reagent, Thermo Scientific), and plasma free cholesterol (Free Cholesterol E kit, Wako Pure Chemicals) and triglyceride levels (L-Type Triglyceride M kit, Wako Pure Chemicals). Levels of IL-6 and TNFα in plasma were analyzed using ELISA MAX Deluxe Sets for mouse IL-6 and TNFα from BioLegend.

### Monocyte recruitment

Monocyte recruitment into atherosclerotic plaques was monitored as described by Randolph and co-workers [40]. Briefly, BM transplanted mice fed the HFCC diet for 17 days were injected intravenously with 250 µl of a solution containing 1.5×10^11^ green fluorescent 0.5 µm beads (Polysciences, Inc.). Circulating monocytes have been shown to take up the fluorescent beads and then enter tissues, including atherosclerosis plaques [50]. Twenty-four hours later, mice were euthanized and atherosclerotic plaques in the aortic sinus were analyzed as described above. Briefly, plaques were stained for lipid with oil red O and subjected to wide-field fluorescence microscopy for fluorescent latex beads. The numbers of fluorescent latex beads in atherosclerotic plaques in the entire aortic sinus section were quantified for each section and normalized to the cross sectional area of the atherosclerotic plaque. Eight sections were analyzed for each mouse.

### Isolation of peritoneal macrophages and in vitro foam cell formation

Resident peritoneal macrophages were isolated by peritoneal lavage from SR-BI^+/+^apoE-hypomorphic or SR-BI^−/−^apoE-hypomorphic mice fed a normal chow diet. Cells were cultured on chambered cover slips in Dulbecco’s modified eagles medium containing 10% fetal bovine serum, 2 mM L-glutamine and penicillin and streptomycin as previously described [3]. Cells were incubated either without or with 100 µg/ml AcLDL (Biomedical Technologies Inc, Stoughton MA USA) for 24 hrs prior to fixation and stained with oil red O and DAPI. Wide field fluorescent images were captured using a Zeiss Axiovert 200 M inverted fluorescence microscope and oil red O staining was quantified as a measure of lipid accumulation as the average pixel intensity/number of DAPI stained nuclei per field of view. Five fields of view were analyzed per sample and the experiment was done three times in triplicate.

### VCAM-1 and ICAM-1 binding

VCAM-1 and ICAM-1 binding assays were done as described in previous studies [51]-[53]. Blood was collected from SR-BI^+/+^apoE-R61^h/h^ or SR-BI^−/−^apoE-R61^h/h^ mice fed a normal chow diet. Red blood cells were lysed. For ICAM-1 binding, leukocytes were incubated with an anti-CD49d antibody (PE conjugate) to identify monocytes, and incubated with soluble recombinant ICAM-1-human IgG fusion, followed by incubation with an Alexa Fluor 488 Goat Anti-Human IgG (H+L) antibody (Invitrogen-Life Technologies Inc, Burlington ON, Canada). For VCAM-1 binding, leukocytes were incubated with anti-CD11b antibody (PE conjugate) to identify monocytes, and incubated with soluble recombinant VCAM-1-human IgG fusion, followed by incubation with Alexa Fluor 488 Goat Anti-Human IgG (H+L) antibody (Invitrogen-Life Technologies Inc, Burlington ON, Canada). All other antibodies for flow cytometry were from eBioscience Inc (San Diego, CA, USA). Recombinant ICAM-1-human IgG and VCAM-1-human IgG fusion proteins were from R&D Systems, distributed by Cedarlane Labs (Burlington, ON, Canada). Samples were analyzed by flow cytometry using a BD FACScalibur flow cytometer. Monocytes were gated as side scatter (SSC) low and either CD11b or CD49d high [54], [55]. Similar results were obtained when mice were fed either normal or HFCC diet.

### Statistical Analysis

Data was subjected to the Shapiro-Wilk test for normality. Those that passed were analyzed by the Student’s T-test. Those that failed the test for normality were analyzed by the Mann-Whitney Rank Sum test. Sigma Plot Statistical analysis software was used. Differences were considered statistically significant when P<0.05.

## References

[1] Zannis VI, Chroni A, Krieger M (2006) Role of apoA-I, ABCA1, LCAT, and SR-BI in the biogenesis of HDL. J Mol Med 84: 276–294. 16501936 10.1007/s00109-005-0030-4

[2] Yancey PG, Bortnick AE, Kellner-Weibel G, de la Llera-Moya M, Phillips MC, et al (2003) Importance of different pathways of cellular cholesterol efflux. Arterioscler Thromb Vasc Biol 23: 712–719. 12615688 10.1161/01.ATV.0000057572.97137.DD

[3] Zhang Y, Ahmed AM, McFarlane N, Capone C, Boreham DR, et al (2007) Regulation of SR-BI-mediated selective lipid uptake in Chinese hamster ovary-derived cells by protein kinase signaling pathways. J Lipid Res 48: 405–416. 17079793 10.1194/jlr.M600326-JLR200

[4] Brundert M, Ewert A, Heeren J, Behrendt B, Ramakrishnan R, et al (2005) Scavenger receptor class B type I mediates the selective uptake of high-density lipoprotein-associated cholesteryl ester by the liver in mice. Arterioscler Thromb Vasc Biol 25: 143–148. 15528479 10.1161/01.ATV.0000149381.16166.c6

[5] Varban ML, Rinninger F, Wang N, Fairchild-Huntress V, Dunmore JH, et al (1998) Targeted mutation reveals a central role for SR-BI in hepatic selective uptake of high density lipoprotein cholesterol. Proc Natl Acad Sci U S A 95: 4619–4624. 9539787 10.1073/pnas.95.8.4619 PMC22539

[6] Out R, Hoekstra M, Spijkers JA, Kruijt JK, van Eck M, et al (2004) Scavenger receptor class B type I is solely responsible for the selective uptake of cholesteryl esters from HDL by the liver and the adrenals in mice. J Lipid Res 45: 2088–2095. 15314100 10.1194/jlr.M400191-JLR200

[7] Rigotti A, Trigatti BL, Penman M, Rayburn H, Herz J, et al (1997) A targeted mutation in the murine gene encoding the high density lipoprotein (HDL) receptor scavenger receptor class B type I reveals its key role in HDL metabolism. Proc Natl Acad Sci U S A 94: 12610–12615. 9356497 10.1073/pnas.94.23.12610 PMC25055

[8] Trigatti B, Rayburn H, Vinals M, Braun A, Miettinen H, et al (1999) Influence of the high density lipoprotein receptor SR-BI on reproductive and cardiovascular pathophysiology. Proc Natl Acad Sci U S A 96: 9322–9327. 10430941 10.1073/pnas.96.16.9322 PMC17781

[9] Mardones P, Quinones V, Amigo L, Moreno M, Miquel JF, et al (2001) Hepatic cholesterol and bile acid metabolism and intestinal cholesterol absorption in scavenger receptor class B type I-deficient mice. J Lipid Res 42: 170–180. 11181745

[10] Braun A, Trigatti BL, Post MJ, Sato K, Simons M, et al (2002) Loss of SR-BI expression leads to the early onset of occlusive atherosclerotic coronary artery disease, spontaneous myocardial infarctions, severe cardiac dysfunction, and premature death in apolipoprotein E-deficient mice. Circ Res 90: 270–276. 11861414 10.1161/hh0302.104462

[11] Covey SD, Krieger M, Wang W, Penman M, Trigatti BL (2003) Scavenger receptor class B type I-mediated protection against atherosclerosis in LDL receptor-negative mice involves its expression in bone marrow-derived cells. Arterioscler Thromb Vasc Biol 23: 1589–1594. 12829524 10.1161/01.ATV.0000083343.19940.A0

[12] Van Eck M, Twisk J, Hoekstra M, Van Rij BT, Van der Lans CA, et al (2003) Differential effects of scavenger receptor BI deficiency on lipid metabolism in cells of the arterial wall and in the liver. J Biol Chem 278: 23699–23705. 12639961 10.1074/jbc.M211233200

[13] Huszar D, Varban ML, Rinninger F, Feeley R, Arai T, et al (2000) Increased LDL cholesterol and atherosclerosis in LDL receptor-deficient mice with attenuated expression of scavenger receptor B1. Arterioscler Thromb Vasc Biol 20: 1068–1073. 10764675 10.1161/01.atv.20.4.1068

[14] Zhang S, Picard MH, Vasile E, Zhu Y, Raffai RL, et al (2005) Diet-induced occlusive coronary atherosclerosis, myocardial infarction, cardiac dysfunction, and premature death in scavenger receptor class B type I-deficient, hypomorphic apolipoprotein ER61 mice. Circulation 111: 3457–3464. 15967843 10.1161/CIRCULATIONAHA.104.523563

[15] Kozarsky KF, Donahee MH, Rigotti A, Iqbal SN, Edelman ER, et al (1997) Overexpression of the HDL receptor SR-BI alters plasma HDL and bile cholesterol levels. Nature 387: 414–417. 9163428 10.1038/387414a0

[16] Wang N, Arai T, Ji Y, Rinninger F, Tall AR (1998) Liver-specific overexpression of scavenger receptor BI decreases levels of very low density lipoprotein ApoB, low density lipoprotein ApoB, and high density lipoprotein in transgenic mice. J Biol Chem 273: 32920–32926. 9830042 10.1074/jbc.273.49.32920

[17] Ueda Y, Royer L, Gong E, Zhang J, Cooper PN, et al (1999) Lower plasma levels and accelerated clearance of high density lipoprotein (HDL) and non-HDL cholesterol in scavenger receptor class B type I transgenic mice. J Biol Chem 274: 7165–7171. 10066776 10.1074/jbc.274.11.7165

[18] Arai T, Wang N, Bezouevski M, Welch C, Tall AR (1999) Decreased atherosclerosis in heterozygous low density lipoprotein receptor-deficient mice expressing the scavenger receptor BI transgene. J Biol Chem 274: 2366–2371. 9891004 10.1074/jbc.274.4.2366

[19] Kozarsky KF, Donahee MH, Glick JM, Krieger M, Rader DJ (2000) Gene transfer and hepatic overexpression of the HDL receptor SR-BI reduces atherosclerosis in the cholesterol-fed LDL receptor-deficient mouse. Arterioscler Thromb Vasc Biol 20: 721–727. 10712397 10.1161/01.atv.20.3.721

[20] Ueda Y, Gong E, Royer L, Cooper PN, Francone OL, et al (2000) Relationship between expression levels and atherogenesis in scavenger receptor class B, type I transgenics. J Biol Chem 275: 20368–20373. 10751392 10.1074/jbc.M000730200

[21] Braun A, Zhang S, Miettinen HE, Ebrahim S, Holm TM, et al (2003) Probucol prevents early coronary heart disease and death in the high-density lipoprotein receptor SR-BI/apolipoprotein E double knockout mouse. Proc Natl Acad Sci U S A 100: 7283–7288. 12771386 10.1073/pnas.1237725100 PMC165867

[22] Karackattu SL, Picard MH, Krieger M (2005) Lymphocytes Are Not Required for the Rapid Onset of Coronary Heart Disease in Scavenger Receptor Class B Type I/Apolipoprotein E Double Knockout Mice. Arterioscler Thromb Vasc Biol. 10.1161/01.ATV.0000158310.64498.ac 15692099

[23] Karackattu SL, Trigatti B, Krieger M (2006) Hepatic lipase deficiency delays atherosclerosis, myocardial infarction, and cardiac dysfunction and extends lifespan in SR-BI/apolipoprotein E double knockout mice. Arterioscler Thromb Vasc Biol 26: 548–554. 16397139 10.1161/01.ATV.0000202662.63876.02

[24] Raffai RL, Dong LM, Farese RV Jr, Weisgraber KH (2001) Introduction of human apolipoprotein E4 "domain interaction" into mouse apolipoprotein E. Proc Natl Acad Sci U S A. 98: 11587–11591. 10.1073/pnas.201279298 PMC58773 11553788

[25] Raffai RL, Weisgraber KH (2002) Hypomorphic apolipoprotein E mice: a new model of conditional gene repair to examine apolipoprotein E-mediated metabolism. J Biol Chem 277: 11064–11068. 11792702 10.1074/jbc.M111222200

[26] Landschulz KT, Pathak RK, Rigotti A, Krieger M, Hobbs HH (1996) Regulation of scavenger receptor, class B, type I, a high density lipoprotein receptor, in liver and steroidogenic tissues of the rat. J Clin Invest 98: 984–995. 8770871 10.1172/JCI118883 PMC507514

[27] Hatzopoulos AK, Rigotti A, Rosenberg RD, Krieger M (1998) Temporal and spatial pattern of expression of the HDL receptor SR-BI during murine embryogenesis. J Lipid Res 39: 495–508. 9548583

[28] Yuhanna IS, Zhu Y, Cox BE, Hahner LD, Osborne-Lawrence S, et al (2001) High-density lipoprotein binding to scavenger receptor-BI activates endothelial nitric oxide synthase. Nat Med 7: 853–857. 11433352 10.1038/89986

[29] Hirano K, Yamashita S, Nakagawa Y, Ohya T, Matsuura F, et al (1999) Expression of human scavenger receptor class B type I in cultured human monocyte-derived macrophages and atherosclerotic lesions. Circ Res 85: 108–116. 10400916 10.1161/01.res.85.1.108

[30] Imachi H, Murao K, Cao WM, Ohyama T, Sato M, et al (2001) Expression of HDL receptor, CLA-1 in human smooth-muscle cells and effect of interferon-gamma on its regulation. Horm Metab Res 33: 389–393. 11507674 10.1055/s-2001-16237

[31] Imachi H, Murao K, Cao W, Tada S, Taminato T, et al (2003) Expression of human scavenger receptor B1 on and in human platelets. Arterioscler Thromb Vasc Biol 23: 898–904. 12649086 10.1161/01.ATV.0000067429.46333.7B

[32] Barth H, Schnober EK, Neumann-Haefelin C, Thumann C, Zeisel MB, et al (2008) Scavenger receptor class B is required for hepatitis C virus uptake and cross-presentation by human dendritic cells. J Virol 82: 3466–3479. 18216094 10.1128/JVI.02478-07 PMC2268490

[33] Zhang Y, Da SilvaJR, Reilly M, Billheimer JT, Rothblat GH, et al (2005) Hepatic expression of scavenger receptor class B type I (SR-BI) is a positive regulator of macrophage reverse cholesterol transport in vivo. J Clin Invest 115: 2870–2874. 16200214 10.1172/JCI25327 PMC1236682

[34] Zhang W, Yancey PG, Su YR, Babaev VR, Zhang Y, et al (2003) Inactivation of macrophage scavenger receptor class B type I promotes atherosclerotic lesion development in apolipoprotein E-deficient mice. Circulation 108: 2258–2263. 14581413 10.1161/01.CIR.0000093189.97429.9D

[35] Van Eck M, Bos IS, Hildebrand RB, Van Rij BT, Van Berkel TJ (2004) Dual role for scavenger receptor class B, type I on bone marrow-derived cells in atherosclerotic lesion development. Am J Pathol 165: 785–794. 15331403 10.1016/S0002-9440(10)63341-X PMC1618614

[36] Yu H, Zhang W, Yancey PG, Koury MJ, Zhang Y, et al (2006) Macrophage apolipoprotein E reduces atherosclerosis and prevents premature death in apolipoprotein E and scavenger receptor-class BI double-knockout mice. Arterioscler Thromb Vasc Biol 26: 150–156. 16269665 10.1161/01.ATV.0000194096.89476.73

[37] Boisvert WA, Spangenberg J, Curtiss LK (1995) Treatment of severe hypercholesterolemia in apolipoprotein E-deficient mice by bone marrow transplantation. J Clin Invest 96: 1118–1124. 7635947 10.1172/JCI118098 PMC185301

[38] Van Eck M, Herijgers N, Yates J, Pearce NJ, Hoogerbrugge PM, et al (1997) Bone marrow transplantation in apolipoprotein E-deficient mice. Effect of ApoE gene dosage on serum lipid concentrations, (beta)VLDL catabolism, and atherosclerosis. Arterioscler Thromb Vasc Biol 17: 3117–3126. 9409301 10.1161/01.atv.17.11.3117

[39] Galkina E, Ley K (2007) Vascular adhesion molecules in atherosclerosis. Arterioscler Thromb Vasc Biol 27: 2292–2301. 17673705 10.1161/ATVBAHA.107.149179

[40] Tacke F, Alvarez D, Kaplan TJ, Jakubzick C, Spanbroek R, et al (2007) Monocyte subsets differentially employ CCR2, CCR5, and CX3CR1 to accumulate within atherosclerotic plaques. J Clin Invest 117: 185–194. 17200718 10.1172/JCI28549 PMC1716202

[41] Getz GS, Reardon CA (2006) Diet and murine atherosclerosis. Arterioscler Thromb Vasc Biol 26: 242–249. 16373607 10.1161/01.ATV.0000201071.49029.17

[42] Lawrie A, Hameed AG, Chamberlain J, Arnold N, Kennerley A, et al (2011) Paigen diet-fed apolipoprotein E knockout mice develop severe pulmonary hypertension in an interleukin-1-dependent manner. Am J Pathol 179: 1693–1705. 21835155 10.1016/j.ajpath.2011.06.037 PMC3181351

[43] Al-Jarallah A, Trigatti BL (2010) A role for the scavenger receptor, class B type I in high density lipoprotein dependent activation of cellular signaling pathways. Biochim Biophys Acta 1801: 1239–1248. 20732452 10.1016/j.bbalip.2010.08.006

[44] Feng H, Guo L, Wang D, Gao H, Hou G, et al (2011) Deficiency of scavenger receptor BI leads to impaired lymphocyte homeostasis and autoimmune disorders in mice. Arterioscler Thromb Vasc Biol 31: 2543–2551. 21836069 10.1161/ATVBAHA.111.234716 PMC3202973

[45] Huber SA, Sakkinen P, Conze D, Hardin N, Tracy R (1999) Interleukin-6 exacerbates early atherosclerosis in mice. Arterioscler Thromb Vasc Biol 19: 2364–2367. 10521365 10.1161/01.atv.19.10.2364

[46] Murphy AJ, Woollard KJ, Hoang A, Mukhamedova N, Stirzaker RA, et al (2008) High-density lipoprotein reduces the human monocyte inflammatory response. Arterioscler Thromb Vasc Biol 28: 2071–2077. 18617650 10.1161/ATVBAHA.108.168690

[47] Roque-Cuellar MC, Sanchez B, Garcia-Lozano JR, Garrido-Serrano A, Sayago M, et al (2012) Expression of CD81, SR-BI and LDLR in lymphocytes and monocytes from patients with classic and occult hepatitis C virus infection. J Med Virol 84: 1727–1736. 22997075 10.1002/jmv.23345

[48] Smythies LE, White CR, Maheshwari A, Palgunachari MN, Anantharamaiah GM, et al (2010) Apolipoprotein A-I mimetic 4F alters the function of human monocyte-derived macrophages. Am J Physiol Cell Physiol 298: C1538–1548. 20219948 10.1152/ajpcell.00467.2009 PMC2889631

[49] Kaplanski G, Marin V, Montero-Julian F, Mantovani A, Farnarier C (2003) IL-6: a regulator of the transition from neutrophil to monocyte recruitment during inflammation. Trends Immunol 24: 25–29. 12495721 10.1016/s1471-4906(02)00013-3

[50] Tacke F, Randolph GJ (2006) Migratory fate and differentiation of blood monocyte subsets. Immunobiology 211: 609–618. 16920499 10.1016/j.imbio.2006.05.025

[51] Feng C, Zhang L, Almulki L, Faez S, Whitford M, et al (2011) Endogenous PMN sialidase activity exposes activation epitope on CD11b/CD18 which enhances its binding interaction with ICAM-1. J Leukoc Biol 90: 313–321. 21551251 10.1189/jlb.1210708 PMC3133440

[52] Lichterfeld M, Martin S, Burkly L, Haas R, Kronenwett R (2000) Mobilization of CD34+ haematopoietic stem cells is associated with a functional inactivation of the integrin very late antigen 4. Br J Haematol 110: 71–81. 10930981 10.1046/j.1365-2141.2000.02130.x

[53] Rose DM, Cardarelli PM, Cobb RR, Ginsberg MH (2000) Soluble VCAM-1 binding to alpha4 integrins is cell-type specific and activation dependent and is disrupted during apoptosis in T cells. Blood 95: 602–609. 10627469

[54] Sunderkotter C, Nikolic T, Dillon MJ, Van Rooijen N, Stehling M, et al (2004) Subpopulations of mouse blood monocytes differ in maturation stage and inflammatory response. J Immunol 172: 4410–4417. 15034056 10.4049/jimmunol.172.7.4410

[55] Woollard KJ, Geissmann F (2010) Monocytes in atherosclerosis: subsets and functions. Nat Rev Cardiol 7: 77–86. 20065951 10.1038/nrcardio.2009.228 PMC2813241

